# Genetic and Physical Interactions between Tel2 and the Med15 Mediator Subunit in *Saccharomyces cerevisiae*

**DOI:** 10.1371/journal.pone.0030451

**Published:** 2012-01-24

**Authors:** Nathalie Grandin, Laetitia Corset, Michel Charbonneau

**Affiliations:** 1 UMR CNRS 5239, Ecole Normale Supérieure de Lyon, IFR128 BioSciences Gerland, Lyon, France; 2 UMR CNRS 6239, Université de Tours, Tours, France; Tulane University Health Sciences Center, United States of America

## Abstract

**Background:**

In budding yeast, the highly conserved Tel2 protein is part of several complexes and its main function is now believed to be in the biogenesis of phosphatidyl inositol 3-kinase related kinases.

**Principal Findings:**

To uncover potentially novel functions of Tel2, we set out to isolate temperature-sensitive (ts) mutant alleles of *TEL2* in order to perform genetic screenings. *MED15*/*GAL11*, a subunit of Mediator, a general regulator of transcription, was isolated as a suppressor of these mutants. The isolated *tel2* mutants exhibited a short telomere phenotype that was partially rescued by *MED15*/*GAL11* overexpression. The *tel2*-*15*mutant was markedly deficient in the transcription of *EST2*, coding for the catalytic subunit of telomerase, potentially explaining the short telomere phenotype of this mutant. In parallel, a two-hybrid screen identified an association between Tel2 and Rvb2, a highly conserved member of the AAA+ family of ATPases further found by *in vivo* co-immunoprecipitation to be tight and constitutive. Transiently overproduced Tel2 and Med15/Gal11 associated together, suggesting a potential role for Tel2 in transcription. Other Mediator subunits, as well as *SUA7*/TFIIB, also rescued the *tel2*-*ts* mutants.

**Significance:**

Altogether, the present data suggest the existence of a novel role for Tel2, namely in transcription, possibly in cooperation with Rvb2 and involving the existence of physical interactions with the Med15/Gal11 Mediator subunit.

## Introduction

*TEL2* is a highly conserved gene that has been found in all eukaryotic organisms examined so far. Before describing the very important and fundamental roles of Tel2 known to date, it is worth briefly describing the chronology of Tel2's history. Tel2 was originally isolated as a potential regulator of telomere length in the budding yeast *Saccharomyces cerevisiae*, but became eventually, over twenty years later, highly suspected, in humans at least but also in the fission yeast *Schizosaccharomyces pombe*, of having nothing to do with telomeres [1]. In the meantime, several, sometimes contradictory, telomere studies on Tel2 from various organisms had accumulated. The Tel2 story begins in 1986, when Lustig and Petes isolated, on the basis of telomere tracts length, two *S. cerevisiae* mutant strains that had abnormally short telomeres and named them *tel1* and *tel2* [2]. Telomeres, specialized nucleoprotein complexes, represent the natural ends of linear chromosomes and their integrity is essential for genome stability. Telomeres protect against unwanted chromosome end-to-end fusions, against degradation by DNA modifying enzymes and prevent chromosome ends from being mistaken for DNA double-strand breaks [3]. The genes corresponding to the short telomere mutants isolated by Lustig and Petes [2], *TEL1* and *TEL2*, were cloned around ten years later [4], [5]. Tel1, as well as its human ortholog, ATM (Ataxia Telangiectasia Mutated), have been since extensively documented as they both play pivotal roles in genome stability as well as in the response of the cell to DNA damage, while Tel2 remained poorly documented for many years following its identification. In fact, Tel1 has a true role in telomere biology in budding yeast as it was recently found to localize at the ends of the shortest telomeres in the cell, an event which then favors telomerase recruitment and telomere re-elongation [6]–[8].

On the other hand, early experiments on *S. cerevisiae* Tel2 revealed that it could bind telomeric DNA, at least *in vitro* [9], [10]. Not long later, probably led by the potential implication of *S. cerevisiae* Tel2 in telomeric functions, several studies on Tel2 from other organisms aiming at looking for telomeric functions were undertaken. Thus, human *TEL2*, also known as *HCLK2*, and *Caenorhabditis elegans TEL2*, also known as RAD-5 or CLK-2, were implicated in the control of telomere length [11]–[13]. Meanwhile, other studies investigated in other directions and, as a consequence, Tel2 was also implicated in the response to DNA damage, in *C. elegans* [12], [14], *S. pombe* [15] and humans [16], [17]. This was also the case in *S. cerevisiae*, as Tel2 was found to physically bind Tel1, an event that was needed to recruit Tel1 at DNA double-strand breaks, as well as Mec1, an ATR ortholog [18], [19]. Given the localization of Tel1 at short telomeres [6], [7], it would be extremely important to know whether Tel2 is needed to recruit Tel1 at short telomeres, as it does at DNA double-strand breaks [18], [19]. In mammals, Tel2 physically associated with -and was required for the stability- of all six mammalian phosphoinositide 3-kinase related kinases (PIKKs), ATM, ATR, DNA-PKcs, mTOR, SMG1 and TRRAP [17]. These PIKKs complexes are important for basic transactions associated with DNA damage signaling/repair and associated cell cycle control, nutrient sensing and cell growth control, degradation of mRNA and control of gene expression, principally [1], [20]. Strikingly, however, the study on mammalian TEL2 failed to reveal any evidence for telomeric phenotypes associated with *TEL2* genetic inactivation [17], in spite of the fact that ATM is pivotal in the response of telomeres to DNA damage [3]. In parallel, in *S. pombe*, Tel2 was found to bind to all three PIKKs, Rad3, Tel1 and Trapp1/2 [20], [21]. The data in humans revealing a role of TEL2 in ATM stability, as seen above [17], might well explain the short telomere phenotype of the original *S. cerevisiae tel2*-*1* mutant [2] as Tel1, the ortholog of ATM, might become unstable in that mutant. In fact, very recently, an *S. cerevisiae* temperature-sensitive *tel2* mutant was found to exhibit depressed levels of Tel1 [22], thus potentially indicating instability of Tel1 as was the case for ATM following inactivation of TEL2 in mammalian cells [17].

Recent studies revealed that budding yeast Tel2 was part of a novel complex, named ASTRA (ASsembly of Tel, Rvb and Atm-like kinase), which contained seven subunits: Tel2, Tti1 and Tti2, defining a PIKK biogenesis complex, the AAA+ ATPase complex Rvb1/Rvb2, the PIKK Tra1 and Asa1 [23]. In addition, other even more recent studies revealed the existence of functional interactions between the Tel2, Tti1 and Tti2 subunits (now known as the TTT complex) of the ASTRA complex and the Rvb1/2, Tah1, Pih1 subunits of the so-called R2TP complex (Rvb1/2 being also part of the ASTRA complex) [23]–[29]. In particular, it is interesting to note that, based on structural analyses, yeast Tel2 acted with the molecular chaperone Hsp90 (part of the R2TP complex) in the maturation of PIKK complexes [29]. It has been proposed that TEL2 might act as a scaffold to coordinate the activities of R2TP/prefoldin-like and HSP90 chaperone complexes during the assembly of the PIKKs [26].

In the present study, we report the isolation and initial characterization of *tel2*-*ts* mutants and their utilization as tools in genetic screenings. Unexpectedly, we have isolated *MED15*, a subunit of the Mediator complex, a general regulator of transcription, as a suppressor of these *tel2*-*ts* mutants.

## Results

### Temperature-sensitive *tel2* mutants

**Figure 1:**
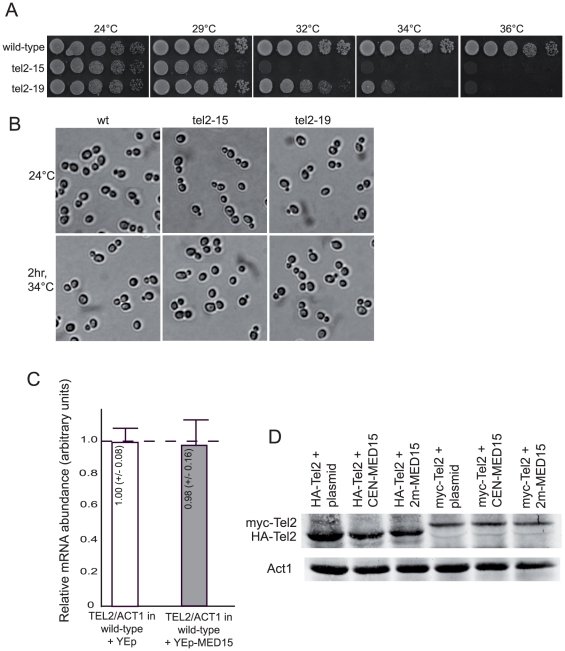
Temperature-sensitive *Saccharomyces cerevisiae tel2* mutants. (**A**) Growth characteristics of the temperature-sensitive *tel2* mutants. Ten-fold serial dilutions (from left to right in each condition) of cultures of the indicated relevant genotype were grown for 3 days on YEPD agar at the indicated temperature and photographed. (**B**) Temperature-sensitive *tel2*-*15* and *tel2*-*19* mutants fail to arrest at a specific cell cycle stage when incubated at the restrictive temperature for growth of 34°C. Cell lysis was evident under the microscope upon extended incubation at restrictive temperatures (not shown). (**C**) Overexpression of *MED15* from a multi-copy plasmid (2 µ, YEp) does not result in an increase in *TEL2* transcription. (**D**) Western blot showing that overexpression of *MED15*, either from a low-copy (CEN) or a multi-copy (2 µ) plasmid, does not result in an increase in the amount of endogenous Tel2 (HA_2_-Tel2 or Myc_2_-Tel2) in the cell (the lanes labeled “HA_2_-Tel2/Myc_2_-Tel2+plasmid” represent the controls with plasmid alone). Endogenous Tel2, tagged with either Myc_2_ or HA_2_ in its N-terminus, was immunoprecipitated from crude extracts with the corresponding monoclonal antibody and Western blotting with the same antibody. Therefore, overexpression of *MED15* does not rescue the *tel2*-*ts* mutants by increasing either *TEL2* transcription or even indirectly by increasing Tel2 protein levels.

We first set out to isolate temperature-sensitive (ts) mutants of *S. cerevisiae TEL2* (see Materials and Methods). We could isolate six such *tel2* alleles. We could distinguish two classes of *tel2* mutants, based on the severity of the temperature-associated growth defects and the capacity to form colonies at 34–37°C (**Figure 1A**) and the nature of the mutations harbored (**Table 1**). Death in these *tel2* mutants was accompanied by cell lysis, which appeared to occur at any of the cell cycle stages. FACS analysis, performed on alpha factor-synchronized populations of *tel2*-*15* and *tel2*-*19* mutant cells, as well as examination in the light microscope, failed to reveal any obvious cell-cycle defect at restrictive temperatures for growth (**Figure 1B** and **data not shown**).

**Table 1:** Sequence analysis of the amino acid changes in the *tel2* mutant proteins.

(All in *tel2*::Kan-MX4 YCp111-pro-*tel2*-tail)
**Growth defects at 34°C:**
Tel2-7: I204V, L303V, S384P, I675V
Tel2-19 and Tel2-26: I204V, L303V, S384P
Tel2-12 (selected as conferring short telomeres): I204V, L303V, S384P
**Tightly thermo-sensitive (growth arrest) at 34°C:**
Tel2-15: I43V, Y90N, M141T, N178S, S206P (+silent L135)
Tel2-25 and Tel2-30: T66S, V134M, E157G, V172A, F306L, H355R, S411R, A627V (+silent V45, V229, T648 in Tel2-25 and silent V45 in Tel2-30)

### The *MED15* Mediator subunit is a suppressor of *tel2*-*ts* mutations

All six *tel2* alleles identified in the present study were used to perform genetic screens in order to try and isolate extragenic suppressors. Only one such gene, namely *GAL11*, was isolated in these genetic screens, besides *TEL2* itself. *GAL11* behaved both as a low-copy and a high-copy suppressor. Thus, *GAL11* was contained in the YCp50#8 centromeric (*CEN4*, *URA3*) plasmid (from the YCp50 genomic library; [30]) isolated as a weak rescuer of *tel2*-*15* at 31°C (see below), as well as in the YEp24#2 multi-copy (episomal, 2 µ, *URA3*) plasmid (from the YEp24 genomic library; [31]), strong rescuer of *tel2*-*26* at 34°C. Restriction analysis identified the *GAL11* gene and surrounding sequences as responsible for the rescue in YCp50#8 and YEp24#2, both around 4.2 kb in length (*GAL11* ORF is 3243 base pairs in length). In addition, *TEL2* itself expressed from a YEp24 library plasmid was isolated twice as a strong rescuer of *tel2*-*19* and *tel2*-*25* at 35 and 33°C, respectively (see below). Gal11, which according to a unified nomenclature should now be referred to as Med15 [32], is a subunit of the yeast Mediator, a large complex required for the recruitment of RNA polymerase II to activated promoters (see, for instance, [33]). We therefore considered the possibility that increased expression of *MED15* might activate the transcription of *TEL2*, thus resulting in increased Tel2 protein levels being responsible for the observed suppression of *tel2*-*ts* growth defects by the YCp50#8 and YEp24#2 plasmids. By Western analysis, the levels of either HA_2_-Tel2 or Myc_2_-Tel2, both expressed under the control of native promoter from the tagged construct integrated at *TEL2* locus, did not vary whether *MED15* was expressed from either the centromeric or multi-copy plasmid, in addition to endogenous Med15, or expressed from endogenous locus only, in cells transformed with plasmid alone (**Figure 1D**). These data therefore suggest that suppression of the thermosensitivity of *tel*-*15* or *tel2*-*26* by YCp50-*MED15* or YEp24*-MED15*, respectively, is not due to increased amounts of *TEL2* protein. In addition, we also verified that the suppression was not due to increased transcription of *TEL2* (**Figure 1C**).

### *TEL2* genetically interacts with other Mediator subunits, as well as with *SUA7*/TFIIB

In parallel with the genetic screenings described above, we performed a two-hybrid screen using Tel2 as the bait. Only one clone scored positive, identifying *S. cerevisiae RVB2* cDNA (see **Appendix S1** in the **Supporting information** and **Figures S1, S2**).

Rvb1/2, two highly conserved members of the AAA+ family of ATPases, are parts of several distinct complexes, namely, the SWR1 and INO80 complexes of chromatin modification, the R2TP complex which, together with the Hsp90 chaperone, regulates the accumulation and stability of snoRNPs, and the ASTRA complex, the function of which is presently unknown [23]–[24], [33]–[35]. In all of these multi-protein complexes, the presence of Rvb1 and that of Rvb2 were reported to be essential for their proper function. In addition, Rvb1/pontin and Rvb2/reptin have been implicated in general transcription, not only in *S. cerevisiae* in which roughly 5% of all genes were found to be deregulated in *rvb2* mutants [36], [37] but also in Vertebrates [38]. In view of the solid and constitutive physical association between Rvb2 and Tel2 and of the genetic interaction between *TEL2* and the *MED15* Mediator subunit, described above, we hypothesized that Tel2 might also function as a general regulator of transcription.

Sua7, budding yeast TFIIB, an essential component of RNA polymerase II [39] has previously been reported to bind Tel2 by two-hybrid [40]. This putative physical interaction, obtained from high-throughput data, has not been retested and cannot therefore be used to draw any valid conclusion. However, this arose our curiosity and we set out to test the potential existence of genetic interactions between *TEL2* and *SUA7*. Interestingly, continuous overexpression (on agar-based semi-solid medium) of *SUA7* under the control of the *GAL1*-*10* promoter resulted in the suppression of *tel2*-*19* and *tel2*-*25* growth defects at 35 and 32°C, respectively (**data not shown**). On the other hand, under the same conditions, overexpression of *RVB2*, also under *GAL1*-*10* promoter control, did not rescue the growth defects in the thermo-sensitive *tel2*-*19* and *tel2*-*25* mutants and, in fact, aggravated these defects (**data not shown**).

**Figure 2:**
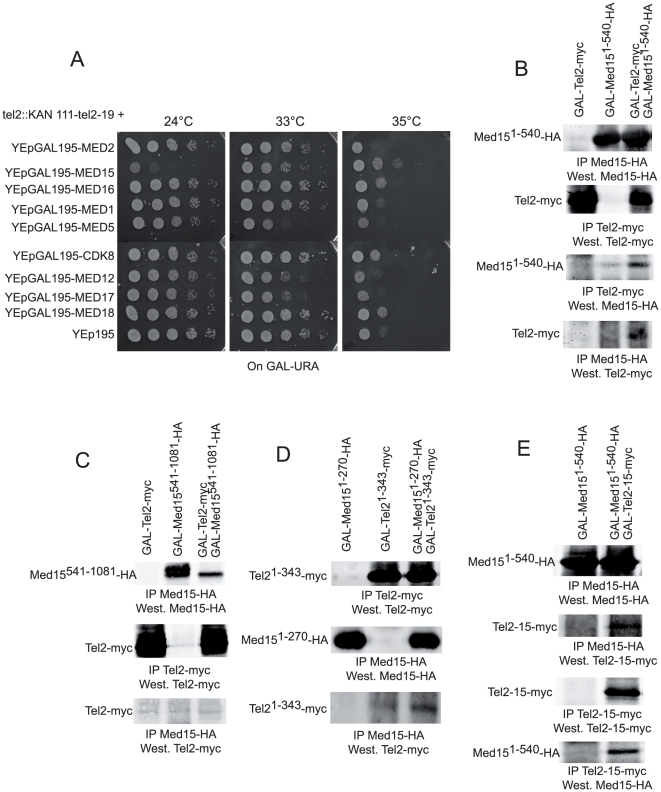
Increased dosage of several Mediator genes partially rescue the temperature sensitivity of the *tel2*-*19* mutant. (**A**) Overexpression of the indicated genes, under the control of the *GAL1*-*10* promoter, was continuously induced by growing on solid media containing galactose as the sole carbon source. Cells transformed with the indicated plasmid were first grown in liquid culture in selective minimal medium containing glucose as the carbon source before being re-streaked on agar-based selective minimal medium containing galactose as the carbon source. Growth was assessed after 3 days at the indicated temperature in the serially diluted cells (ten-fold dilutions from left to right in each condition). At 35°C, *MED16*, *CDK8* and *MED18* overexpression rescued *tel2*-*19*, although slightly less efficiently than *MED15*. (**B**) Tel2 and Med15 physically interact *in vivo*. Putative physical interactions between Tel2-Myc_3_ and Med15-HA_2_ were measured upon immunoprecipitation (IP) with anti-HA or anti-Myc monoclonal antibodies, followed by Western blotting (West.), from crude extracts from strains having transiently overexpressed the indicated constructs after incubation in galactose-based liquid selective media, for 2 hr at 29°C (same conditions for panels B–E). All experiments were conducted in parallel in strains expressing both constructs and in strains with the single constructs to take into account possible background signals. Full length Tel2-Myc_3_ specifically binds the first half of Med15 (Med15^1–540^-HA_2_) in both directions, that is to say that a positive signal was detected whether one or the other of the two proteins was immunoprecipitated. (**C**) In contrast, the second half of Med15 (Med15^541–1081^-HA_2_) did not associate *in vivo* with full length Tel2-Myc_3_. (**D**) Finally, the first quarter of Med15 (Med15^1–270^-HA_2_) efficiently bound the first half of Tel2 (Tel2^1–343^-HA_2_). (**E**) At the maximal permissive temperature for growth for *tel2*-*15* of 29°C, the Tel2-15-Myc_3_ protein still physically interacted with Med15^1–540^-HA_2_, as was also the case at 34°C (data not shown).

Next, to see whether *tel2*-*ts* rescue by *MED15* might reflect a general property of Mediator, we constructed plasmids to express one of several additional Mediator subunits under the control of the strong, inducible *GAL1*-*10* promoter in a multi-copy (2 µ) plasmid. Among the *MED1*, *MED2*, *MED5*, *MED12*, *MED16*, *MED17*, *MED18* and *CDK8* genes chosen for this experiment, we found that overexpression of *MED16*, *MED18* and *CDK8*, in addition to that of *MED15*, as described above, resulted in a modest rescue of *tel2*-*19*-*ts* growth defects at 35°C, all slightly less efficient than that by *MED15* (**Figure 2A**).

### The Med15 Mediator subunit has physical affinities with Tel2

The genetic interaction between *MED15* and *TEL2*, described above, prompted us to look for possible physical interactions between the two proteins, interactions that are frequently suspected of taking place under similar circumstances. Therefore, we assessed possible Tel2-Med15 physical interactions under conditions of increased expression by using a *GAL1*-*10* promoter-controlled inducible system. Such a system in which the duration of induction of the promoter in galactose-based liquid culture medium is controlled is currently used in genome-wide analyses because it presents the advantage compared to other systems of assessing *in vivo* interactions between proteins in their native configuration. Upon induction of the *GAL1*-*10* promoter for 2 hr at 29°C, we could readily detect *in vivo* physical interactions between Tel2-Myc_3_ and Med15-HA_2_ (**Figure 2**). Thus, full length Tel2-Myc_3_ was found to specifically bind the first half of Med15 (Med15^1–540^-HA_2_), but not the second half of Med15 (Med15^541–1081^-HA_2_) (**Figure 2B, C**). Additional restriction of the expressed proteins showed that the first quarter of Med15 (Med15^1–270^-HA_2_) efficiently bound the first half of Tel2 (Tel2^1–343^-HA_2_) (**Figure 2D**). Therefore, given the genetic suppression of *tel2*-*ts* mutants by *MED15*, as well as the physical affinity between Med15 and Tel2, it is tempting to speculate that the Rvb2-Tel2 module might play an important role during transcription. The previously established physical interaction between Tel2 and Sua7/TFIIB [40] supports this view.

Finally, under transient overexpression conditions, Med15^1–540^-HA_2_ still physically associated with the temperature-sensitive Tel2-15-Myc_3_ protein at either 29°C or 34°C (**Figure 2E**). Tel2-15 was chosen for these experiments because it confers a tighter ts phenotype than Tel2-19. Note that all five mutations in Tel2-15 (**Table 1**) lie in the part of Tel2 (amino acids 1–343) that is relevant for interaction with Med15 (**Figure 2D**). One can therefore reasonably conclude that these mutations do not affect association with Med15.

### Short telomeres of *tel2* mutants rescued by *MED15* overexpression

**Figure 3:**
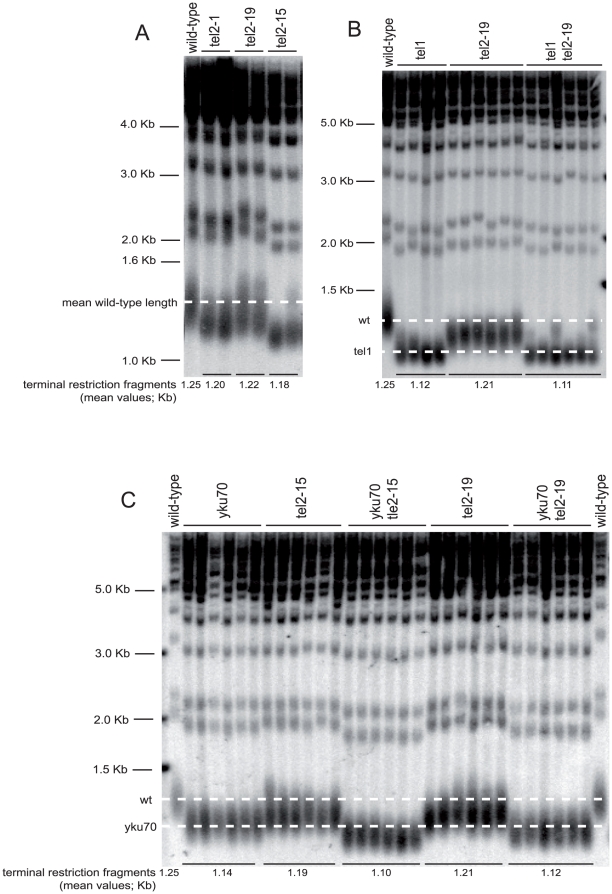
The temperature-sensitive *tel2*-*15* and *tel2*-*19* mutants exhibit short telomeres. (**A**) *tel2*-*15* confers a more severe telomere length defect than *tel2*-*19*, compared here with the previously described *tel2*-*1* mutant [2], all grown at 24°C. (**B**) The shortened telomeres in the *tel2*-*19* mutant were not furthered shortened when combined with a *tel1* null mutation. These strains were grown at 29°C. (**C**). In contrast, both the *tel2*-*19 yku70* null and *tel2*-*15 yku70* null double mutants, grown here at 24°C, exhibited shorter telomeres than either one of the corresponding single mutants. Average telomere length (corresponding to the DNA smear migrating at around 1.3 kb) of *tel2* mutant strains with the indicated relevant genotype, grown for at least 22 days to allow telomere length to attain a stable value, was detected by Southern blotting with a TG_1–3_ telomeric P^32^-labeled probe. The dashed horizontal lines, drawn between the mean length values of two or more wild-type (wt) or mutant strains, as indicated in the margin, allow to get a better appreciation of the variations in telomere length in the mutants.

Prior to isolation of the temperature-sensitive *tel2* mutants described above, we had been searching for *tel2* mutants with altered telomere length (see Materials and Methods), and found one of them (out of 200 hundreds screened mutants), *tel2*-*12*, that exhibited shortened telomeres. The mutations in *tel2*-*12* were later found to be identical to those in the *tel2*-*19* mutant (**Table 1**) and later found to confer temperature sensitivity. Upon further analysis of these mutants, we found that both the *tel2*-*15* and *tel2*-*19* mutants exhibited telomere shortening, just like the *tel2*-*12* mutant, when grown at either permissive, 24°C, (**Figure 3A**) or semi-temperature for growth of 29°C (**Figure 3B**). To further document the telomere length deregulation occurring in the *tel2*-*ts* mutants, we additionally constructed *tel2*-*ts*-based double mutants. Importantly, we observed the absence of an additional effect on telomere length when the *tel1* null and *tel2*-*19* mutations were combined (*tel1* null also confers telomere shortening; [2]; **Figure 3B**), as expected from the previous observation that Tel1 and Tel2 function in the same pathway of telomere length regulation [2]. We also observed that telomeres in the *tel1*Δ *yku70*Δ double mutants were shorter than in the two corresponding single mutants (**Figure S3**). This indicated that *tel1*Δ cells have telomeres that can get even shorter when they are in combination with an additional mutation, thus serving as a positive control for the present situation in which the *tel2* mutation fails to further shorten telomeres in the *tel1* mutant background (Figure 3B). This point had been previously established by a study reporting that the *tel1 hdf1*/*yku70* null double mutant had telomeres shorter than either of the two single *tel1* and *hdf1* single mutants and that the double mutant lost telomeres at an accelerated rate compared with the single mutants [41]. In contrast, combining the *tel2*-*19* (or *tel2*-*15*) and *yku70* null mutations (*yku70* null also confers telomere shortening; [42] provoked an additive effect on telomere shortening (**Figure 3C**). This finding was expected since previous data showed that Tel1 and Yku70 are in separate genetic pathways for telomere length regulation [41] while Tel1 and Tel2 are in the same pathway [2].

**Figure 4:**
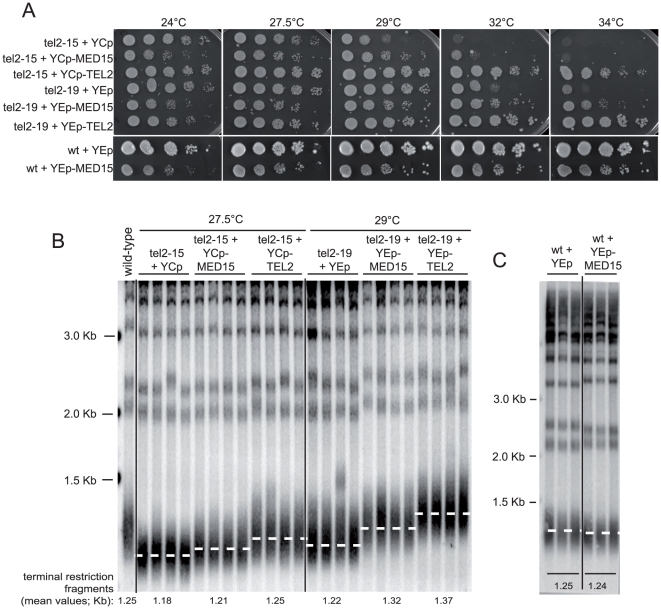
*MED15*, coding for a subunit of Mediator, found in the present study to be an extragenic suppressor of *tel2*-*ts* mutations, also partially rescues their telomere length defect. (**A**) The temperature-sensitive *tel2*-*15* and *tel2*-*19* mutants, as well as a wild-type (wt) strain,were re-transformed, here, with the *MED15* or *TEL2* plasmids, either low-copy (1 or 2 copies) *CEN* (YCplac) or multi-copy 2 µ (YEplac), both originally isolated from the genetic screens performed in the present study, or with the corresponding empty plasmids (YCp or YEp), and their growth characteristics evaluated after spotting ten-fold dilutions (from left to right in each condition) on selective, -Ura, minimal medium at the indicated temperatures. YCp-*MED15* rescued *tel2*-*15* at up to 29°C, while YEp-*MED15* rescued *tel2*-*19* at up to 34°C. (**B**) Overexpression of *MED15*, from the plasmids described above, in A, partially rescued the short telomere phenotype of the *tel2*-*15* and *tel2*-*19* mutant strains. Compare the extent of the rescue with that provided by *TEL2* overexpression. Telomere length was measured as described in the legend to Figure 3 after the strains had been propagated at 27.5°C (*tel2*-*15*) or 29°C (*tel2*-*19*) on minimal medium for 30 days. (**C**) Overexpression of *MED15* had no elongation effect on telomere length in wild-type cells, grown here at 29°C. In fact, a slight shortening of telomeres was observed following *MED15* overexpression compared with expression of vector alone.

Interestingly, overexpression of *MED15* not only partially rescued the temperature sensitivity of the *tel2*-*15* and *tel2*-*19* mutants (**Figure 4A**), but also partially rescued their short telomere phenotype at semi-permissive temperatures for growth (**Figure 4B**; see also **Figure 4C** for the wild-type control). At the permissive temperature of 24°C, the telomere length defect of the *tel2*-*ts* mutants, which is already present (Figure 3A), was also partially rescued by overexpression of *MED15* (**data not shown**). We noted that overexpression of *MED15* caused growth defects at 24°C in the *tel2*-*ts* mutants (**Figures 2A** **,** **4A**) and at all temperatures tested in the wild type (**Figure 4A**). While this did not prevent rescue of the *tel2*-*ts* at higher temperatures, this may be important to keep in mind. We also note that overexpression of other Mediator subunits did not result in such a toxic effect (**Figure2A**). Therefore, manipulating the levels of Med15 in wild-type cells may cause toxic effects by potentially titrating out other essential components. We do not have at the moment the exact explanation for this phenomenon and its elucidation will require future analysis.

### Tel2 controls the transcription of *EST2*/telomerase

The *tel2*-*15* mutant described above has been recently reported to also affect telomere length at semi-permissive temperature for growth [22]. In the *tel2*-*15* mutant, the levels of Tel1 were dramatically depressed [22], thus providing a rational explanation for the fact that this mutant exhibits short telomeres ([22]; present data), *tel1* null mutant also exhibiting short telomeres [2].

**Figure 5:**
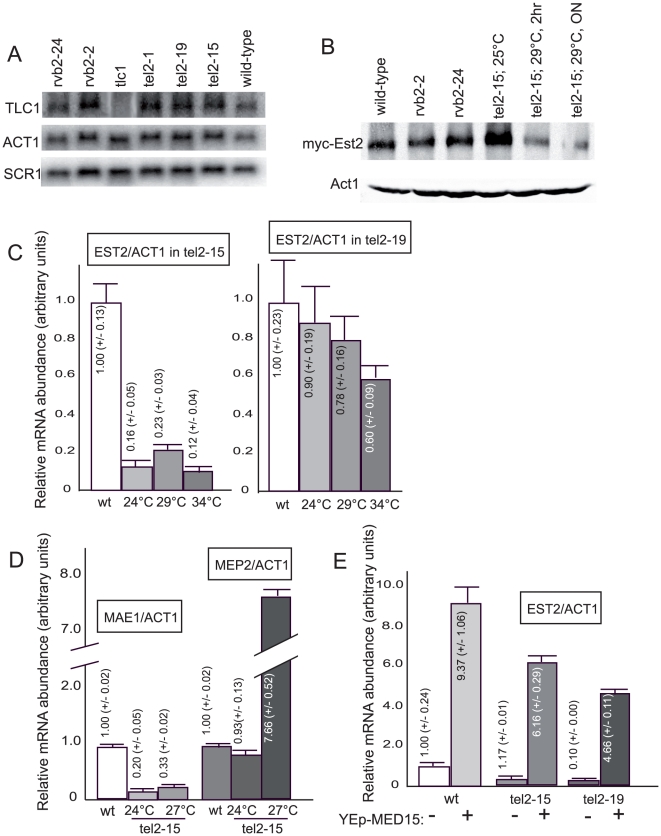
Tel2 controls transcription of *EST2*/telomerase (but not that of the telomerase RNA subunit *TLC1*) and is regulated by the Med15 Mediator subunit. (**A**) Northern blot analysis of endogenous *TLC1* RNA levels indicates an absence of deregulation in the indicated *tel2* mutants. *rvb2* mutants that exhibit slight and stable telomere shortening (NG, MC, submitted for publication) were also used in that experiment. A *tlc1* null strain was used to ascertain that the highlighted band indeed corresponds to TLC1 RNA. *ACT1* and *SCR1* RNA levels were measured to serve as loading controls. (**B**) Immunoprecipitation-Western experiments aiming at assessing Myc_18_-Est2 levels (construct integrated at *EST2* genomic locus, under the control of native promoter) indicate that *EST2*/telomerase levels are depressed in the *tel2*-*15* ts mutant strain grown at the semi-permissive temperature for growth of 29°C for 2 hr or overnight (ON). The *rvb2*-*2* and *rvb2*-*24* mutants (NG, MC, submitted for publication) were used as controls. (**C**) Levels of *EST2* mRNA, coding for the protein subunit of budding yeast telomerase, were measured relative to those of *ACT1*, in wild-type cells (wt, normalized to 1.0) and in the temperature-sensitive *tel2*-*15* (**left panel**) and *tel2*-*19* (**right panel**) mutants. The mean values ± standard error correspond to 4 experiments performed with the *tel2*-*15* mutant and 5 experiments with the *tel2*-*19* mutant, each sample being performed in triplicate. (**D**) *MAE1* and *MEP2* mRNA levels in the *tel2*-*15* ts mutant, at the indicated temperatures. Results are from two experiments, each sample being performed in triplicate. (**E**) Continuous overexpression of the *MED15* Mediator subunit, achieved by transforming the strains of the indicated relevant genotype with an episomal (2 µ) vector harboring *MED15* ORF flanked by upstream and downstream natural genomic sequences, resulted in a dramatic increase in *EST2* mRNA levels. Data are from two experiments using the wild type and the two mutants, each sample being performed in triplicate.

However, based on the data above, the short telomere phenotype of the *tel2*-*15* and *tel2*-*19* mutants might additionally be due to defects in telomerase transcription potentially resulting from alterations of functional interactions between Tel2 and Med15 and/or between Tel2 and Rvb2 in these mutants. These observations next prompted us to measure telomerase transcription in these *tel2* mutants. First, we set out to measure levels of *TLC1* RNA, the template subunit for *S. cerevisiae* telomerase, in the *tel2*-*15* and *tel2*-*19* mutants. No significant difference could be seen in *TLC1* RNA levels in these mutants when compared with the wild type (**Figure 5A**). In contrast to the situation with *TLC1*, the transcription of *EST2*, coding for the protein subunit of telomerase, was clearly diminished in the *tel2*-*15* mutant grown at semi-permissive temperature and even at permissive temperature for growth and only modestly diminished in the *tel2*-*19* mutant (**Figure 5C**), consistent with the fact that the *tel2*-*15* ts mutant is tighter than the *tel2*-*19* ts mutant (Figures 1A). In agreement with the data on *EST2* transcription reported above, we observed that the level of endogenous Myc_18_-Est2 was depressed in strains harboring the *tel2*-*15* mutation compared with the *TEL2* ^+^ strain (**Figure 5B**). We noted that, inconsistently, in this particular experiment the level of Myc_18_-Est2 at the permissive temperature of 24°C was even higher in the *tel2*-*15* mutant than in the wild-type strain (**Figure 5B**). To understand this phenomenon, we examined all available data on the subject. In fact, in other experiments, one of which using the *tel2*-*19* mutant is shown in **Figure S4**, the level of Myc_18_-Est2 was found to be similar to that in the wild type at 24°C. In another experiment, the level of Myc_18_-Est2 in *tel2*-*15* at 24°C was lower than that in the wild type (**data not shown**). Therefore, we do not know yet whether there is a mode of regulation of Est2 particular to the *tel2*-*15* mutant, perhaps, which we do not understand yet and which might be an indirect effect. The *tel2*-*15* mutant is more severe than the *tel2*-*19* mutant and may be deregulated in a more unpredictable manner at permissive temperatures. Nevertheless, it is clear that Myc_18_-Est2 levels were greatly perturbed in both mutants at 29°C (**Figures 5B** **, S4**). Incidentally, we found that the *tel2*-*15* and *tel2*-*19* mutations did not provoke a reduction in *TEL1* trancript levels (**data not shown**). Therefore, the depression of Tel1 protein levels observed in the *tel2*-*15* mutant [22] seems to be due to uncorrect assembly of the Tel1 PIKK kinase [17] rather than to repressed transcription, as previously suggested [22].

As mentioned above, Rvb2 has been found to control the expression of around 5% of the *S. cerevisiae* genes [36]. If Tel2 controlled general transcription in association with Rvb2, mutations in either one should result in a similar gene expression signature. To test this possibility, we selected two genes, *MAE1* and *MEP2*, the expression of which have been previously shown to be up-regulated and down-regulated by Rvb2, respectively [36], [37] and measured their levels of expression in the *tel2*-*15* mutant. Interestingly, the same trend in the expression of *MAE1* and *MEP2* was found in the *tel2*-*15* mutant compared with that in the *tih2*-*160* mutant, a temperature-sensitive mutant of *RVB2* [37]. Indeed, *MAE1* mRNA levels were severely depressed in the *tel2*-*15* mutant, while, on the opposite, *MEP2* mRNA levels were dramatically increased in that same mutant (**Figure 5D**), a figure similar to that found in the *tih2*-*160*/*rvb2* mutant [37].

These observations next prompted us to evaluate the impact of overexpressing *MED15* on *EST2* transcription. Interestingly, overexpressing the *MED15* subunit of Mediator under the control of its natural promoter resulted in a dramatic increase in *EST2*/telomerase transcription (**Figure 5E**).

We presumed that *est2* null mutant cells were not temperature-sensitive during the period of time, around 75 generations, they were still alive prior to reaching telomeric senescence [43], as this has never been reported in the literature. However, to make sure that this was the case, we directly tested it and found that, indeed, the *est2* null mutant cells prior to senescence were only moderately temperature-sensitive (**Figure S5**) unlike the *tel2*-*15* and *tel2*-*19* mutants which were markedly temperature-sensitive, as seen above. Moreover, the growth defects in the *est2*Δ mutant could be explained by the damage generated by telomere erosion (see the legend to **Figure S5**). These observations strongly suggested that the *tel2*-*ts* phenotype was not simply due the diminution of *EST2*/telomerase transcription. In addition, we note that the *tel2*-*ts* mutants arrest at various stages during the cell cycle, as seen above, while the telomerase/*est2*Δ mutants arrest at the G2/M border [43]. In fact, recent genome wide data suggest that the *tel2*-*ts* mutants are likely to harbor cumulative defects in the transcription of several genes, thereby leading, directly or indirectly, to the depressed levels of endogenous Est2 observed here. Indeed, one of the *tel2*-*ts* mutants uncovered here, *tel2*-*7* (see mutations, **Table 1**) exhibited genetic interactions, under the form of synthetic growth defects, with numerous and various mutations ([44]; see also the BioGRID, Toronto, at http://thebiogrid.org; the *tel2*-*7* mutant was provided by us to these authors). We therefore conclude that the short telomere phenotype of *tel2*-*ts* mutants is not solely due to a diminution in telomerase transcription, as proposed above and in agreement with previous observations [22].

## Discussion

The present study does not bring much more information concerning the possible telomeric functions of *S. cerevisiae* Tel2, with the exception of a possible general role in *EST2*/telomerase transcription (but also in the transcription of other genes). However, unexpectedly, we find that the *MED15* subunit of Mediator (a general regulator of transcription) is a genetic suppressor of the *tel2*-*ts* mutations isolated in the present study. This genetic interaction was further documented by the finding of an *in vivo* interaction between Tel2 and Med15. Overexpression of other Mediator subunits, as well as of *SUA7*/TFIIB, also rescued the *tel2*-*ts* growth defects. We also report here the existence of a physical association between Tel2 and Rvb2. Potentially, based on the already known role of Rvb2 in controlling transcription, the Tel2-Rvb2 interaction might represent a necessary basis for the functional interactions between Tel2 and Med15.

In addition to the recently discovered role of human RUVBL1/2 in mRNA stability (at least those of the PIKKs) *via* the SMG1-mediated nonsense-mediated mRNA decay (NMD) pathway [28], there is overwhelming evidence that they regulate transcription when functioning in the SRCAP, TIP60 and INO80 chromatin-modifying complexes [34], [38]. Most likely, *S. cerevisiae* Rvb1/2 perform similar tasks in regulating transcription when part of the respective homologous complexes, SWR1, NuA4 and INO80 [34]. In view of the present results, it is interesting to speculate that the *S. cerevisiae* Rvb2-Tel2 module might play a major role in the expression of multiple genes, as already demonstrated for Rvb2 [34]–[37], including of *EST2*/telomerase, as shown here. This is further supported by the observation that Med15-HA_2_ physically interacted with Rvb2-Myc_3_ when both were transiently overproduced (NG, MC, unpublished data). Interestingly, genetic inactivation of pontin/hRUVBL1 or of reptin/hRUVBL2 was recently found to result in decreases in mRNA levels of *hTERT*, the catalytic subunit of human telomerase, effect partly exerted through binding of reptin to *hTERT* proximal promoter [45]. Moreover, overexpression of reptin was observed in primary gastric cancer specimens [45]. In a separate study, on colon cancer cells, RUVBL2 was also found to regulate *hTERT* transcription [46]. Regarding *EST2*/telomerase expression in *S. cerevisiae*, it is worth mentioning that its levels of mRNA, as well as those of other genes coding for telomeric proteins, namely Est1, Est3, Stn1 and Ten1, were found to be regulated by the NMD pathway [47], thus establishing a potential link between the Rvb2-Tel2 module and the NMD pathway, as shown for human RUVBL1/2 [28].

The putative role of Tel2 in transcription might be parallel and distinct from its role in the biogenesis of the PIKKs complexes. Because *MED15* overexpression rescued the growth defects of the *tel2*-*ts* mutants, and Med15 can physically associate with Tel2, we further speculate that the function of Tel2 in transcription uncovered here probably depends on physical interactions with the Mediator *via* its Med15 subunit. A possible clue stems from the observation of genetic interactions between Tel2 and Sua7, uncovered here, as well as between Sua7 and Med15/Gal11 [48]. *SUA7* encodes yeast TFIIB, a general transcription factor required for the initiation of transcription by RNA polymerase II [39]. Mediator's general function is to recruit RNA polymerase II at sites of active transcription [32], [33]. Med15/Gal11 was found to partially suppress deletions of *GAL4* and *GCN4*, encoding two general transcriptional activators [48]. These authors proposed that transcriptional activators work by raising the local concentration of the limiting factor Med15/Gal11, and that Med15/Gal11 works by recruiting Mediator and Taf14-containing transcription factors like TFIID and SWI/SNF and by competing general repressors like Ssn6-Tup1 off the target promoters [48]. Potentially, transcriptional activators might recruit the Mediator, *via* Med15, to the chosen site of transcription, while simultaneously recruiting Sua7 *via* Tel2. The role of Tel2 in these transactions might merely be to assist in chromatin remodeling in cooperation with Rvb1/2 in order to facilitate physical interactions between Sua7 and the Mediator.

Another possible clue to explain the events observed here is related to telomere position effect (TPE), a phenomenon that reversibly silences genes situated close to telomeres [49]. Interestingly, in *S. cerevisiae*, both Tel2 and Med15 have been reported to affect telomere position effect. Thus, the *tel2-1* mutation reduced TPE but had no detectable effect on silencing of *HML*a or *HMR*a, the silent mating type cassettes [5]. However, to our knowledge, the reasons for the implications of Tel2 in TPE have not been uncovered since, except for the proposal that Tel2 may be required for chromatin assembly at telomeres and elsewhere in the genome [5]. On the other hand, a mutation in *GAL11*/*MED15* or, on the contrary, its overexpression, both affected TPE and telomere structure [50]. Moreover, a mutation in *GAL11*/*SDS4*/*MED15* was found to strongly suppress a *rap1*-induced silencing defect at the telomeres and the *HMR* locus [51]. In fact, very recently, it was established that the Mediator directly associated with heterochromatin at telomeres, thereby influencing the exact boundary between active and inactive chromatin [52]. Mutations in Mediator subunits also resulted in increased levels of H4K16 acetylation near telomeres and in desilencing of subtelomeric genes, an effect that appeared to be distinct from the role of Mediator as a co-trancriptional activator [52]. Therefore, the interactions between Tel2 and Med15 described in the present work might theoretically have something to do with their effect on telomere structure. Further work will be required to test these hypotheses, which might be complex and difficult to experimentally approach. For the moment, we have ruled out a role for Tel2 in telomere end protection, as our *tel2*-*ts* mutants did not activate the DNA damage checkpoint, unlike mutants defective in telomere protection such as *cdc13*-*1*, for instance (NG, MC, unpublished data). However, more subtle defects of telomere structure resulting from *TEL2* inactivation, such as those discovered in early studies [5], might be responsible for the functional interactions with *MED15* uncovered here.

As stressed above, Tel2 has long been an enigmatic protein, implicated in a myriad of apparently unrelated biological processes in various organisms. However, recent studies have uncovered what appears to be the main function of Tel2, namely in PIKK biogenesis, and other more recent studies have begun to unravel these mechanisms at the molecular level. However, a recent analysis showed that *C. elegans* CLK-2/TEL2 depletion did not phenocopy PIKK kinase depletion and, in addition, implicated CLK-2/TEL2 in multiple developmental and cell cycle related processes [53]. The genetic approach developed here has provided new clues to further understand Tel2 functions and, unexpectedly, orientate the research on Tel2 towards directions that had not been suspected before. The present data will hopefully serve as a starting point for further exploring the role of this pivotal protein, Tel2, in potentially novel mechanisms of regulation of transcription.

## Materials and Methods

### Yeast strains, plasmids and inducible overexpression by galactose

Yeast strains used in this study were derivatives of BF264-15Daub (*ade1 his2 leu2-3,112 trp1-1a ura3Dns*), as described previously [54]. Yeast cultures were grown at the indicated temperatures in YEP (1% yeast extract, 2% bacto-peptone, 0.005% adenine, 0.005% uracile) supplemented with 2% glucose (YEPD), sucrose or galactose, or in selective minimal medium. All strains were made isogenic by back crossing at least five times against our genetic background. Strain origins, prior to back crossing, were as follows. The *rvb2*::*KanMX4*/*RVB2*, *tel2*::*KanMX4*/*TEL2*, *est2*::*KanMX4*/*EST2*, *tel1*::*KanMX4* and *yku70*::*KanMX4* strains were purchased at Euroscarf (Frankfurt, Germany). The *tlc1*::*TRP1* strain was from the Gottschling laboratory.

Two-hybrid experiments using the pACT2 and pAS2 vectors and pACT1 cDNA library were performed as described previously [55].

All constructs were made by using Polymerase Chain Reaction (PCR) to adapt the relevant restriction sites to the sequence of the genes and details of the constructs will be made available upon request. To confirm the two-hybrid interaction between Tel2 and Rvb2 and delineate the domains of interaction, we opted for a transient overexpression system, currently used in genome-wide analyses, which presents the advantage of measuring *in vivo* interactions of proteins in their native configuration. To have accurate control of the extent of expression, we used the galactose-inducible *GAL1*-*10* promoter and activated it only in a transient manner to avoid possible deleterious effects of heavy overexpression. *RVB2* was tagged with a 2 HA-6 His (hereafter referred to as HA_2_ because an anti-HA monoclonal antibody was used throughout) and *TEL2* with a 3 Myc (Myc_3_) epitope tag, both at their 3′ end. Induction of genes placed under the control of the *GAL1*-*10* promoter was done by transferring cells growing in liquid culture in glucose-containing medium into galactose-containing liquid medium after several washes by centrifugation. In experiments involving expression of protein parts under *GAL1*-*10* promoter control, a supplementary methionine residue was added in front of the truncated sequence (if starting from places other than the natural initiating ATG) to initiate efficient translation.

### Western blotting and immunoprecipitation

Techniques for block and release experiments, flow cytometry analysis (FACS), cell extract preparation, immunoprecipitation and immunoblotting (analyzed using an Odyssey Imager) have been described previously [54], [55]. Mouse monoclonal anti-HA raw ascites fluid 16B12 (BabCO) and mouse monoclonal anti-HA 12CA5 antibody (Roche Diagnostics) were used for immunoprecipitation and imunoblotting, respectively. Myc-tagged proteins were visualized after immunoprecipitation and Western blotting with monoclonal anti-Myc antibody 9E10 (Roche Diagnostics). Anti-actin antibody, clone C4, was from MP Biomedicals.

### Telomere length measurement

To analyze telomere length, genomic DNAs were prepared, separated in a 0.9% agarose gel (in TBE) run in TBE buffer overnight and, after denaturation, transferred and hybridized with a 270 base pair TG_1–3_^32^P-labeled telomeric probe as described previously [55]. Following digestion of genomic DNA with *Xho*I, to cut within the Y′ regions of chromosomes, telomere tracts of wild-type cells appear as a broad band of ∼1.1–1.3 kb which represents the average length of most chromosomes. Results were analyzed using an FLA-5100 Fuji phosphoimager and the ImageGauge software.

### Northern blotting and analysis of transcription by RT-PCR

For analysis of TLC1 endogenous levels, total RNA was first isolated from yeast cell cultures according to standard procedures. Northern blot analysis was conducted according to classical techniques using a P^32^-labeled probe specific for *TLC1* sequences. A *tlc1* null strain was used in all experiments to attest for the specificity of the detected signals.

The relative quantification of mRNA was performed with a quantitative RT-PCR assay. Gene-specific primers were designed using Universal Probe Library Assay Design Center (Roche Applied Science) as primer software. Two micrograms of RNA were reverse transcribed using the first strand cDNA synthesis kit from Fermentas, with gene specific primer for *EST2* and random hexamers as primers for the other genes. cDNAs were then diluted to a final concentration of 10 ng/µl in sterile H_2_O and amplified using a BioRad Opticon instrument. Amplification was performed in 20 µl of reaction mix, containing 5 µl of diluted cDNA and 15 µl Mesa Green qPCR Master SYBR Green I (Eurogentec), together with forward and reverse primers. PCR was performed according to a two-step protocol: 3 min at 95°C, followed by 45 cycles comprising each 10 s at 95°C (denaturation) and 30 s at 60°C (annealing/extension). Quantitative data of the samples were obtained using the BioRad CFX Manager software. All cDNA samples were assayed in triplicates. We chose the *ACT1* housekeeping gene as the endogenous normalizer because its expression was constant.

### Mutagenesis of *TEL2*

Mutagenesis of *TEL2* to isolate temperature-sensitive was performed by mutagenic PCR coupled to the so-called gap repair method to generate in vivo the plasmids having copied the in vitro-generated mutant alleles, as described below. To mutagenize *TEL2*, the 2064 base pair (bp)-long *TEL2* ORF plus ∼215 bp upstream of the ATG and ∼270 bp downstream of the stop codon was amplified by error-prone PCR under the following conditions. The concentration of dNTPs was either kept as in standard conditions (200 µM each) or one of the four dNTP concentration changed to 0.5–1.0 mM, those of the other three being kept at 200 µM, and, in both cases, the concentration of MgCl_2_ was changed from 1.5 to 3.0 or 4.0 mM and 0.5 mM MnCl_2_ added to the reaction. Standard *Taq* polymerase and PCR buffer (Promega) were used. Following a 30-cycle amplification, the mutated fragments were transformed into a *tel2*::*KanMX4* strain in which the deletion was complemented by wild-type *TEL2* borne on a *CEN*-*URA3* (YClacp33) plasmid, together with a centromeric, *LEU2*-based (YCplac111), plasmid carrying *TEL2* ORF plus the same flanking regions and made linear by digestion with *Nru*I and *Stu*I, endogenous sites located ∼50 base pairs post-initiating ATG and ∼140 base pairs downstream of the stop codon, respectively. After shuffling out the wild-type *TEL2* allele on 5-FOA medium (which counter-selects for *URA3* in the plasmid), colonies growing at 24°C were replica plated at 33–37°C to identify thermosensitive colonies. We could isolate six such *tel2* alleles. All six alleles were sub-cloned from the initial centromeric vector into a single copy vector (*LEU2*-based, YIp128) that was subsequently integrated at the *LEU2* locus and re-transformed into the original *tel2* null YCplac33-*TEL2* strain, followed by 5-FOA counter-selection.

To isolate *tel2* mutants potentially deregulated in telomere length control, the initial processes were similar to those exposed above. However, at the step next after shuffling out of the wild type *TEL2* allele on 5-FOA medium, transformants were picked out randomly from the plates, propagated for ∼20 days at 24°C to attain telomere length equilibrium and selected by Southern blot analysis, as described above.

### Cell viability assays

The viability of cells previously grown in liquid was determined by performing and analyzing the so-called “drop tests”. To do this, cells from exponential growth cultures were counted with a hematocytometer and the cultures were then serially diluted by 1/5^th^ or 1/10^th^ and spotted onto either selective plates or YEPD non-selective plates, as required, and incubated at the desired temperature for 2–3 days before being photographed.

## Supporting Information

Figure S1 **(see Appendix S1): Physical interactions between Tel2 and Rvb2.** (**A**) Endogenous Rvb2 and Tel2 physically associate even when telomerase access to the telomeres is restricted or even totally prevented. Immunoprecipitation-Western (IP-West.) blotting experiments on endogenous HA_2_-*RVB2* and Myc_2_-*TEL2* in an otherwise wild type background (2^nd^ lane) or in strains containing either an *est1* (3^rd^ lane), a *yku70* (4^th^ lane) or *est1 yku70* null mutation (5^th^ lane) to restrict telomerase access to the telomeres either in the G1 and early S phases (*yku70* null), late S, G2 and M phases (*est1* null), or in all cell cycle phases (*est1* null *yku70* null) [2]. The 1^st^ lane is a control with no Myc_2_-*TEL2* expression to assess for HA_2_-Rvb2 background. (**B**) Overproduced full length Tel2-Myc_3_ physically interacts with full length Rvb2-HA_2_, in both directions. Tel2-Myc_3_ was specifically detected in Rvb2-HA_2_ immunoprecipitate (left panel, top gel) and reciprocally (right panel, bottom gel) over the background; compare, in each panel, lanes 3 (strain harboring both constructs) with lanes 1 and 2 (strains harboring the single constructs). Overexpression of the constructs, here and below in C, under the control of the inducible *GAL1*-*10* promoter was for 2 hr at 29°C. (**C**) Overproduced Tel2 and Rvb2 still physically associate in the absence of Tel1 (which has been previously reported to physically associate with Tel2; [3]), also creating a situation in which telomerase access to the telomeres is restricted. The association between Rvb2-HA_2_ and Tel2-Myc_3_ could be detected whether the immunoprecipitation (IP) was directed against Tel2-Myc_3_ (left panel) or, on the opposite, against Rvb2-HA_2_ (right panel), and the Western blotting (West.) realized with anti-HA and anti-Myc monoclonal antibody, respectively, as indicated below each gel. (**D**) Tel2 did not physically associate with amino acids 420 to 740 of Est2, comprising the entire catalytic domain when they were overproduced, for 2 hr at 29°C, under the control of the inducible *GAL1*-*10* promoter in a high-copy vector. These experiments were performed using strains expressing either full length Tel2 (Tel2-Myc_3_, **left panel**), or Tel2-Myc_3_ first half (**middle panel**) or Tel2-Myc_3_ second half (**right panel**), as well as Est2's catalytic domain (Est2^420–740^-HA_2_).
(EPS)

Figure S2 **(see Appendix S1): Delineating the Tel2 and Rvb2 fragments needed for physical interaction.** (**A**) The first half of Tel2, Tel2-(1–343)-Myc_3_, but not its second half, Tel2-(344–688)-Myc_3_, had affinity with full length Rvb2-HA_2_. The two panels show the same thing in two distinct experiments. Note, in bottom gels, that the first 343 amino acids of Tel2 migrated at an apparent molecular weight of around 33 kD, while roughly the same number of amino acids in the second part, the last 345 ones, migrated at an apparent molecular weight of around 48 kD. (**B**) Full length Tel2-Myc_3_ physically interacted with the first half of Rvb2, Rvb2-(1–235)-HA_2_, in both directions, as shown in **left and middle panels**, but barely with its second half, Rvb2-(236–470)-HA_2_ (**right panel**). (**C**) Rvb2-(1–118)-HA_2_, the first half of Rvb2's first half, clearly associated with Tel2 first half, Tel2-(1–343)-Myc_3_, while Rvb2-(119–235)-HA_2_, the second half of Rvb2's first half, did only faintly. (**D**) Rvb2-(1–235)-HA_2_ had no clear physical interactions with the first two halves of Tel2 first half, namely Tel2-(1–171)-HA_2_ and Tel2-(172–343)-HA_2_, implying that sequences in both of these two Tel2 fragments are essential for association with Rvb2. (**E**) Schematic of Tel2-Rvb2 interactions showing in grey the regions of Tel2 and Rvb2 implicated here in physical association between the two proteins.
(EPS)

Figure S3 **The** ***tel1*** **null** ***yku70*** **null double mutant exhibited shorter telomeres than the two single mutants** ***tel1*** **Δ and** ***yku70*** **Δ.** The mutants were grown (at 29°C) sufficiently long to harbor telomeres having attained length equilibrium. See the legend to Figure 3 for more technical detail.
(EPS)

Figure S4 **Immunoprecipitation-Western experiments, performed as described in the legends to** **Fig. 5B** **and S1, aiming at assessing Myc_18_-Est2 levels (construct integrated at** ***EST2*** **genomic locus, under the control of native promoter) indicate that** ***EST2*** **/telomerase levels are depressed in the** ***tel2*** **-** ***19*** **ts mutant strain grown at the semi-permissive temperatures for growth of 29°C for 2 hr or restrictive temperature of 34°C for 4 hr, but not at the permissive temperature of 24°C, as indicated.**
(EPS)

Figure S5 **(A)** ***est2*** **null mutants prior to telomeric senescence are only moderately temperature-sensitive.** Therefore, the strong ts phenotype of the *tel2*-*15* and *tel2*-*19* mutants cannot be due to the diminution of *EST2* transcript levels in these mutants. As soon as *EST2* has been genetically inactivated, telomeric DNA damage accumulates, thus presumably provoking the slight growth defect seen at all temperatures tested in these cells compared with the wild type. Note that at elevated temperatures, e. g. 36°C, cells divide faster thus accelerating accumulation of damage and progression through senescence and provoking these growth defects. Finally, the absence of telomerase provokes a general destabilization of the telomeres that will eventually activate the DNA damage checkpoint and slow down cell cycle progression. Ten-fold serial dilutions (from left to right in each condition) of cultures of the indicated relevant genotype were grown for 3 days on YEPD agar at the indicated temperature and photographed. (**B**) Southern blot analysis of telomere length in the *tel2*-*ts* and pre-senescing *est2*Δ mutants grown at 29°C.
(EPS)

Appendix S1 **Rvb2-Tel2 association detected in a two hybrid screen.**
(DOC)
