# Supplementary material for: Genetic and Physical Interactions between Tel2 and the Med15 Mediator Subunit in Saccharomyces cerevisiae
Source: PLoS One. 2012 Jan 24;7(1):e30451. doi: 10.1371/journal.pone.0030451 (PMC3265489; doi:10.1371/journal.pone.0030451)
Supplement: Appendix S1 — Rvb2-Tel2 association detected in a two hybrid screen. (DOC) [file pone.0030451.s006.doc]

**SUPPORTING INFORMATION**

**Appendix S1**

Rvb2-Tel2 association detected in a two hybrid screen

In parallel with the genetic screenings described above, we performed a two-hybrid screen using Tel2 as the bait. Only one clone, pACT1-cDNA#8, was isolated, among around 15,000 transformants, on the basis of allowing growth of the Y190-pAS2-*TEL2* strain on histidine-, leucine- and trytophane-lacking medium supplemented with 10-30 mM of 3-AT. After rechecking the phenotype, using the recovered plasmid transformed back into the original strain, sequencing identified the cDNA as that of *S*. *cerevisiae RVB2*. Sequences corresponding to amino acids 103 to 299 (total Rvb2 ORF is 471 residues) were contained in pACT1-cDNA#8. The two-hybrid interaction between Tel2 and Rvb2 is in agreement with the fact that both proteins were identified within the same complex, the ASTRA complex [1].

To confirm this two-hybrid interaction between Tel2 and Rvb2, we expressed HA_2_-*RVB2* and Myc_2_-*TEL2*, from their respective chromosomal locus. Association between the two proteins was clearly detected using monoclonal anti-Myc to immunoprecipitate endogenous Myc_2_-Tel2 (Figure S1A, left two lanes). Moreover, this association remained constant in intensity throughout the cell cycle, as assessed in cells synchronized with the alpha factor pheromone (data not shown).

To confirm the *in vivo* interaction between Tel2 and Rvb2 and delineate the domains of interaction, we opted for a transient overexpression system, currently used in genome-wide analyses, which presents the advantage of assessing *in vivo* interactions of proteins in their native configuration. To have accurate control of the extent of expression, we used the galactose-inducible *GAL1*-*10* promoter and activated it only in a transient manner to avoid possible deleterious effects of heavy overexpression. In this system, full length Tel2-Myc_3_ efficiently co-precipitated with full length Rvb2-HA_2_, whether it was Tel2 or Rvb2 which was immunoprecipitated (Figure S1B).

In a recent study, we have shown the existence of a weak physical interaction between Rvb2 and Est2, the catalytic subunit of telomerase (NG, MC, submitted for publication). We therefore wanted to know, here, whether the association between Tel2 and Rvb2 might take place at the telomeres in a telomerase-dependent manner. The experiments shown in Figure S1A (right third lanes; first lane is the control without Myc_2_-Tel2) established that endogenous Rvb2 and Tel2 physically associated in strains in which loading of telomerase on telomeres was partially or totally compromised (*est1* and *est1* *yku70* null mutants, respectively; [2]). Tel1, the budding yeast ATM-like kinase, was recently shown to physically interact with Tel2 [3]. As shown in Figure S1C, transiently overproduced Rvb2-HA_2_ and Tel2-Myc_3_ still physically associated together in a *tel1* null background.

Since Tel2 readily associates with Rvb2 *in vivo*, as seen above, and Rvb2 with telomerase/Est2 (NG, MC, submitted for publication), we next wondered whether physical association between Tel2 and Est2/telomerase could also be detected. No physical interaction between HA_2_-Tel2 and Myc_18_-Est2 (both expressed from their genomic locus under native promoter control) could be detected at any stage of the cell cycle in cells synchronized in G1 phase by alpha factor (data not shown). Even with the higher expression levels allowed by the *GAL1*-based transient overexpression system, no physical interaction between Tel2-Myc_3_, either full length (Figure S1D, left panel) or first half (Figure S1D, middle panel) or second half (Figure S1D, right panel) and Est2^420-740^-HA_2_ could be detected, while, in contrast, in the same system, physical interaction between Rvb2-HA_2_ and Est2^420-740^-Myc_3_ or Est2^1-420^-Myc_3_ could be readily detected (NG, MC, submitted for publication).

We could demonstrate that, roughly, the first halves of the *TEL2* and *RVB2* proteins were responsible for the observed physical association and that the physical interaction could be detected whether immunoprecipitation was against one or the other protein (Figure S2). More precisely, the first 118 residues of Rvb2 (total ORF is 471 residues) were responsible for most of the interaction with residues 1 to 343 of Tel2 (total ORF is 688 residues). Importantly, we observed that Tel2-(1-171) and Tel2-(172-343) did not physically associate with Rvb2’s first 234 amino acids, thus indicating the requirement of Tel2’s amino acids in both of these (1-171) and (172-343) fragments for association with Rvb2 (Figure S2).

Interestingly, the thermo-sensitive Tel2-15-Myc_3_ and Tel2-19-Myc_3_ mutant proteins still physically interacted with Rvb2-HA_2_ at the restrictive temperature of 34°C, when both were overproduced in the inducible *GAL1*-*10* system (data not shown).

References

1. Shevchenko A, Roguev A, Schaft D, Buchanan L, Haberman B, et al. (2008) Chromatin central: towards the comparative proteome by accurate mapping of the yeast proteomic environment. Genome Biol 9: R167.

2. Chan A, Boulé JB, Zakian VA (2008) Two pathways recruit telomerase to *Saccharomyces cerevisiae* telomeres. PLoS Genet 4: e1000236.

3. Anderson CM, Korkin D, Smith DL, Makovets S, Seidel JJ, et al. (2008) Tel2 mediates activation and localization of ATM/Tel1 kinase to a double-strand break. Genes Dev 22: 854-859.
